# Supplementary material for: Maternity and family leave experiences among female ophthalmologists in the United States
Source: PLoS One. 2023 Apr 25;18(4):e0277376. doi: 10.1371/journal.pone.0277376 (PMC10129012; doi:10.1371/journal.pone.0277376)
Supplement: S1 Fig — This is a copy of the survey administered to participants in the study. (DOCX) [file pone.0277376.s001.docx]

Maternity Leave in Ophthalmology

Start of Block: Intro Part 1

Are you a currently practicing/on temporary or permanent leave/disability or retired ophthalmologist in the United States?

- Yes (1)
- No (2)

Q2 Have you taken family medical or maternity leave for the birth or adoption of a child in your post-medical school career (i.e. during residency, fellowship, or practicing years)?

- Yes (1)
- No (2)

End of Block: Intro Part 1

Start of Block: Intro Part 2

Q16 Current position

- Ophthalmology Resident (1)
- Ophthalmology Fellow (2)
- Practicing Ophthalmologist (3)
- Ophthalmologist on leave/disability (4)
- Retired Ophthalmologist (5)
- Other (6)

Q17 How many years in practice?

- Still in training (1)
- Years in practice (2) __________________________________________________

Q18 Average hours worked per week:

- <20 (1)
- 20-40 (2)
- 41-60 (3)
- 61-80 (4)
- >80 (5)

Q443 If you pursued fellowship training, write in your area of specialty below: (if not applicable write NA)

________________________________________________________________

Q152 How many birth events/adoptions have you had since graduating medical school? (multiples count as one birth event for purposes of our study)

- 1 (1)
- 2 (2)
- 3 (3)
- 4 (4)
- 5 or more (5)

Q153 Had someone recommended delaying family planning at some point in your training or medical career?

- Yes (1)
- No (2)

Q154 Do you feel that your career impacted your family planning?

- Definitely not (1)
- Probably not (2)
- Might or might not (3)
- Probably yes (4)
- Definitely yes (5)

End of Block: Intro Part 2

Start of Block: First Child

Q155 In what year did you take your first family/maternity leave after medical school?

________________________________________________________________

Q156 How old were you when you took your first family/maternity leave after medical school?

________________________________________________________________

Q157 First family/maternity leave: Choose option that best applies

- Pregnancy with no intervention (1)
- Intervention assisted pregnancy (2)
- Adoption (3)

Display This Question:

If First family/maternity leave: Choose option that best applies = Intervention assisted pregnancy

Q226 If your pregnancy required fertility interventions, did you receive adequate support for time off from work for appointments/procedures?

- Extremely inadequate time off/support (1)
- Somewhat inadequate time off/support (2)
- Neutral (3)
- Mostly adequate time off/support (4)
- Extremely adequate time off/support (5)

Q158 First family/maternity leave: Which best describes your practice setting at the time of leave?

- Resident in training (1)
- Fellow in training (2)
- Solo practice (3)
- Private practice ≤5 physicians (4)
- Private practice >5 physicians (5)
- Hospital based practice (6)
- Academic practice (7)
- Private equity group practice (8)

Q159 First family/maternity leave: Did your practice/workplace have a maternity leave policy in place? (not including FMLA)

- Yes (1)
- No (2)
- Unsure (3)

Q161 First family/maternity leave: Were you provided with adequate information about family and maternity leave, and was the leave policy at your workplace easy to understand?

- Extremely inadequate (1)
- Somewhat inadequate (2)
- Neither adequate nor inadequate (3)
- Somewhat adequate (4)
- Extremely adequate (5)

Q162 First family/maternity leave: How many weeks were available to you?

________________________________________________________________

Q163 First family/maternity leave: How many weeks did you take off?

________________________________________________________________

Q206 First family/maternity leave: How many weeks of leave would you have liked to receive?

________________________________________________________________

Q165 First family/maternity leave: Source of leave days: (you may select more than one)

- Sick leave (1)
- Vacation days (2)
- Paid time off (3)
- Specified maternity leave days (4)
- Other (5) __________________________________________________

Q166 First family/maternity leave: Regarding pay during leave, which describes your situation best?

- I did not receive any pay during leave (1)
- I received partial pay during leave (2)
- I received full pay during leave (3)
- Unable to recall (4)
- Other (5)

Display This Question:

If First family/maternity leave: Regarding pay during leave, which describes your situation best? = I received partial pay during leave

Q167 First family/maternity leave: How many weeks were paid?

________________________________________________________________

Display This Question:

If First family/maternity leave: Regarding pay during leave, which describes your situation best? = I did not receive any pay during leave

Or First family/maternity leave: Regarding pay during leave, which describes your situation best? = I received partial pay during leave

Or First family/maternity leave: Regarding pay during leave, which describes your situation best? = Unable to recall

Or First family/maternity leave: Regarding pay during leave, which describes your situation best? = Other

Q168 First family/maternity leave: Did you experience any financial hardship during maternity leave due to change in compensation?

- Yes (1)
- No (2)
- Not applicable (3)
- I cannot recall (4)

Q169 First family/maternity leave: If applicable, did you experience a sizable loss in your clinical production bonus during leave?

- Yes (1)
- No (2)
- Not applicable (3)

Q189 First family/maternity leave: Upon return from maternity/family leave did you experience a reduction in hours worked per week compared to that prior to leave?

- Yes (fill in approx new hours worked below) (4) __________________________________________________
- No (5)
- Not applicable (6)

Display This Question:

If First family/maternity leave: Upon return from maternity/family leave did you experience a reduct... = Yes (fill in approx new hours worked below)

Q193 First family/maternity leave: If applicable, why did you work less hours/week after your family/maternity leave?

- Personal choice (1)
- Determined by employer (2)
- Other (4)
- Not applicable (5)

Q225 First family/maternity leave: Regarding breast/chestfeeding, which best describes your situation:

- I chose to breast/chestfeed at some point in my postpartum period (inclusive or exclusive) (1)
- I wanted to breast/chestfeed but was unable to (2)
- I chose not to breast/chestfeed (3)
- I prefer not to answer (4)

Display This Question:

If First family/maternity leave: Regarding breast/chestfeeding, which best describes your situation: = I chose to breast/chestfeed at some point in my postpartum period (inclusive or exclusive)

Q170 First family/maternity leave: If you chose to breastfeed/chestfeed, were you accommodated by your employer to allow adequate time for milk expression?

- Extremely inadequate (1)
- Moderately inadequate (2)
- Slightly inadequate (3)
- Neither adequate nor inadequate (4)
- Slightly adequate (5)
- Moderately adequate (6)
- Extremely adequate (7)
- Not applicable (8)

Display This Question:

If First family/maternity leave: Regarding breast/chestfeeding, which best describes your situation: = I chose to breast/chestfeed at some point in my postpartum period (inclusive or exclusive)

Q171 First family/maternity leave: Were you made to feel guilty or penalized for time missed for milk expression?

- Yes (1)
- Maybe (2)
- No (3)
- Not applicable (4)

Display This Question:

If First family/maternity leave: Regarding breast/chestfeeding, which best describes your situation: = I chose to breast/chestfeed at some point in my postpartum period (inclusive or exclusive)

Q172 First family/maternity leave: If applicable, do you feel your job impacted the duration of your breastfeeding in a negative way?

- No (1)
- Maybe (2)
- Yes (3)
- Not applicable (4)

Q173 First family/maternity leave: Did you feel supported by colleagues and administration during maternity leave and upon return to work?

- Definitely yes (1)
- Probably yes (2)
- Probably not (3)
- Definitely not (4)

Q174 First family/maternity leave: Did you consider leaving your job after the birth of your child?

- Yes (1)
- No (2)
- I did leave my job (3)
- Unable to recall (4)

Q175 First family/maternity leave: Did you suffer with post-partum depression, anxiety, obsessive compulsive disorder or other mental health disorders in the post-partum period?

- Yes (1)
- Maybe (2)
- No (3)

Q176 First family/maternity leave: What option did you pursue for childcare for the majority of the first year of life?

- Nanny/home childcare (1)
- Relative or family (2)
- Daycare facility (3)
- Stay at home spouse (4)
- I stayed at home to watch my child (5)
- Other (6) __________________________________________________

Q177 First family/maternity leave: Did you feel a greater sense of burnout upon return to work?

- Yes (1)
- Maybe (2)
- No (3)

Q178 First family/maternity leave: Select all that apply: I experienced...

- Derogatory comments by colleagues or staff (1)
- Financial pressure from coworkers / institution (2)
- Termination from my position (3)
- Delay in partnership due to leave (4)
- Anger from patients regarding rescheduled appointments (5)
- Continued pressure to answer emails / perform ancillary duties (6)
- Continued expectation to participate in academic endeavors i.e. submit manuscripts, mentor trainees (7)
- Pressure to return to work earlier than planned (8)

Q179 First family/maternity leave: Did your experience during your first leave impact future family planning decisions?

- Definitely yes (1)
- Probably yes (2)
- Might or might not (3)
- Probably not (4)
- Definitely not (5)

Q212 First family/maternity leave: Did your partner take family leave?

- Yes (1)
- No (2)
- Not applicable (4)

Q181 First family/maternity leave: What was your overall level of satisfaction with your family/maternity leave experience?

- Very unsatisfied (1)
- Somewhat unsatisfied (2)
- Neither unsatisfied nor satisfied (3)
- Somewhat satisfied (4)
- Very satisfied (5)

End of Block: First Child

Start of Block: Free Response

Q218 Please provide any other comments you have concerning your experience with family/maternity leave in ophthalmology.

________________________________________________________________

End of Block: Free Response

Start of Block: Second Child**

Q228 In what year did you take your 2nd family/maternity leave after medical school?

________________________________________________________________

Q229 How old were you when you took your 2nd family/maternity leave after medical school?

________________________________________________________________

Q230 Second family/maternity leave: Choose option that best applies

- Pregnancy with no intervention (1)
- Intervention assisted pregnancy (2)
- Adoption (3)

Display This Question:

If Second family/maternity leave: Choose option that best applies = Intervention assisted pregnancy

Q231 If your pregnancy required fertility interventions, did you receive adequate support for time off from work for appointments/procedures?

- Extremely inadequate time off/support (1)
- Somewhat inadequate time off/support (2)
- Neutral (3)
- Mostly adequate time off/support (4)
- Extremely adequate time off/support (5)

Q348 For my second leave, my employer/benefits were the same as the first leave. (same pay circumstances, same number of allowed weeks off)

- Yes (1)
- No (2)

Display This Question:

If For my second leave, my employer/benefits were the same as the first leave. (same pay circumstanc... = No

Q232 Second family/maternity leave: Which best describes your practice setting at the time of leave?

- Resident in training (1)
- Fellow in training (2)
- Solo practice (3)
- Private practice ≤5 physicians (4)
- Private practice >5 physicians (5)
- Hospital based practice (6)
- Academic practice (7)
- Private equity group practice (8)

Display This Question:

If For my second leave, my employer/benefits were the same as the first leave. (same pay circumstanc... = No

Q233 Second family/maternity leave: Did your practice/workplace have a maternity leave policy in place? (not including FMLA)

- Yes (1)
- No (2)
- Unsure (3)

Display This Question:

If For my second leave, my employer/benefits were the same as the first leave. (same pay circumstanc... = No

Q234 Second family/maternity leave: Were you provided with adequate information about family and maternity leave, and was the leave policy at your workplace easy to understand?

- Extremely inadequate (1)
- Somewhat inadequate (2)
- Neither adequate nor inadequate (3)
- Somewhat adequate (4)
- Extremely adequate (5)

Display This Question:

If For my second leave, my employer/benefits were the same as the first leave. (same pay circumstanc... = No

Q235 Second family/maternity leave: How many weeks were available to you?

________________________________________________________________

Q236 Second family/maternity leave: How many weeks did you take off?

________________________________________________________________

Q237 Second family/maternity leave: How many weeks of leave would you have liked to receive?

________________________________________________________________

Q238 Second family/maternity leave: Source of leave days: (you may select more than one)

- Sick leave (1)
- Vacation days (2)
- Paid time off (3)
- Specified maternity leave days (4)
- Other (5) __________________________________________________

Display This Question:

If For my second leave, my employer/benefits were the same as the first leave. (same pay circumstanc... = No

Q239 Second family/maternity leave: Regarding pay during leave, which describes your situation best?

- I did not receive any pay during leave (1)
- I received partial pay during leave (2)
- I received full pay during leave (3)
- Unable to recall (4)
- Other (5)

Display This Question:

If Second family/maternity leave: Regarding pay during leave, which describes your situation best? = I received partial pay during leave

Q240 Second family/maternity leave: How many weeks were paid?

________________________________________________________________

Display This Question:

If Second family/maternity leave: Regarding pay during leave, which describes your situation best? = I did not receive any pay during leave

Or Second family/maternity leave: Regarding pay during leave, which describes your situation best? = I received partial pay during leave

Or Second family/maternity leave: Regarding pay during leave, which describes your situation best? = Unable to recall

Or Second family/maternity leave: Regarding pay during leave, which describes your situation best? = Other

Q241 Second family/maternity leave: Did you experience any financial hardship during maternity leave due to change in compensation?

- Yes (1)
- No (2)
- Not applicable (3)
- I cannot recall (4)

Q242 Second family/maternity leave: If applicable, did you experience a sizable loss in your clinical production bonus during leave?

- Yes (1)
- No (2)
- Not applicable (3)

Q243 Second family/maternity leave: Upon return from maternity/family leave did you experience a reduction in hours worked per week compared to that prior to leave?

- Yes (fill in approx new hours worked below) (4) __________________________________________________
- No (5)
- Not applicable (6)

Display This Question:

If Second family/maternity leave: Upon return from maternity/family leave did you experience a reduc... = Yes (fill in approx new hours worked below)

Q244 Second family/maternity leave: If applicable, why did you work less hours/week after your family/maternity leave?

- Personal choice (1)
- Determined by employer (2)
- Other (4)
- Not applicable (5)

Q245 Second family/maternity leave: Regarding breast/chestfeeding, which best describes your situation:

- I chose to breast/chestfeed at some point in my postpartum period (inclusive or exclusive) (1)
- I wanted to breast/chestfeed but was unable to (2)
- I chose not to breast/chestfeed (3)
- I prefer not to answer (4)

Display This Question:

If Second family/maternity leave: Regarding breast/chestfeeding, which best describes your situation: = I chose to breast/chestfeed at some point in my postpartum period (inclusive or exclusive)

Q246 Second family/maternity leave: If you chose to breastfeed/chestfeed, were you accommodated by your employer to allow adequate time for milk expression?

- Extremely inadequate (1)
- Moderately inadequate (2)
- Slightly inadequate (3)
- Neither adequate nor inadequate (4)
- Slightly adequate (5)
- Moderately adequate (6)
- Extremely adequate (7)
- Not applicable (8)

Display This Question:

If Second family/maternity leave: Regarding breast/chestfeeding, which best describes your situation: = I chose to breast/chestfeed at some point in my postpartum period (inclusive or exclusive)

Q247 Second family/maternity leave: Were you made to feel guilty or penalized for time missed for milk expression?

- Yes (1)
- Maybe (2)
- No (3)
- Not applicable (4)

Display This Question:

If Second family/maternity leave: Regarding breast/chestfeeding, which best describes your situation: = I chose to breast/chestfeed at some point in my postpartum period (inclusive or exclusive)

Q248 Second family/maternity leave: If applicable, do you feel your job impacted the duration of your breastfeeding in a negative way?

- No (1)
- Maybe (2)
- Yes (3)
- Not applicable (4)

Q249 Second family/maternity leave: Did you feel supported by colleagues and administration during maternity leave and upon return to work?

- Definitely yes (1)
- Probably yes (2)
- Probably not (3)
- Definitely not (4)

Q250 Second family/maternity leave: Did you consider leaving your job after the birth of your child?

- Yes (1)
- No (2)
- I did leave my job (3)
- Unable to recall (4)

Q251 Second family/maternity leave: Did you suffer with post-partum depression, anxiety, obsessive compulsive disorder or other mental health disorders in the post-partum period?

- Yes (1)
- Maybe (2)
- No (3)

Q252 Second family/maternity leave: What option did you pursue for childcare for the majority of the first year of life?

- Nanny/home childcare (1)
- Relative or family (2)
- Daycare facility (3)
- Stay at home spouse (4)
- I stayed at home to watch my child (5)
- Other (6) __________________________________________________

Q253 Second family/maternity leave: Did you feel a greater sense of burnout upon return to work?

- Yes (1)
- Maybe (2)
- No (3)

Q254 Second family/maternity leave: Select all that apply: I experienced...

- Derogatory comments by colleagues or staff (1)
- Financial pressure from coworkers / institution (2)
- Termination from my position (3)
- Delay in partnership due to leave (4)
- Anger from patients regarding rescheduled appointments (5)
- Continued pressure to answer emails / perform ancillary duties (6)
- Continued expectation to participate in academic endeavors i.e. submit manuscripts, mentor trainees (7)
- Pressure to return to work earlier than planned (8)
- Other (9) __________________________________________________

Q255 Did the circumstances of this leave impact your future family planning?

- Definitely yes (1)
- Probably yes (2)
- Might or might not (3)
- Probably not (4)
- Definitely not (5)

Q256 Second family/maternity leave: Did your partner take family leave?

- Yes (1)
- No (2)
- Not applicable (4)

Q257 Second family/maternity leave: What was your overall level of satisfaction with your family/maternity leave experience?

- Very unsatisfied (1)
- Somewhat unsatisfied (2)
- Neither unsatisfied nor satisfied (3)
- Somewhat satisfied (4)
- Very satisfied (5)

End of Block: Second Child**

Start of Block: Third Child **

Q349 In what year did you take your 3rd family/maternity leave after medical school?

________________________________________________________________

Q350 How old were you when you took your 3rd family/maternity leave after medical school?

________________________________________________________________

Q351 Third family/maternity leave: Choose option that best applies

- Pregnancy with no intervention (1)
- Intervention assisted pregnancy (2)
- Adoption (3)

Display This Question:

If Third family/maternity leave: Choose option that best applies = Intervention assisted pregnancy

Q352 If your pregnancy required fertility interventions, did you receive adequate support for time off from work for appointments/procedures?

- Extremely inadequate time off/support (1)
- Somewhat inadequate time off/support (2)
- Neutral (3)
- Mostly adequate time off/support (4)
- Extremely adequate time off/support (5)

Q353 For my 3rd leave, my employer/benefits were the same as the prior leave. (same pay circumstances, same number of allowed weeks off)

- Yes (1)
- No (2)

Display This Question:

If For my 3rd leave, my employer/benefits were the same as the prior leave. (same pay circumstances,... = No

Q354
Third family/maternity leave: Which best describes your practice setting at the time of leave?

- Resident in training (1)
- Fellow in training (2)
- Solo practice (3)
- Private practice ≤5 physicians (4)
- Private practice >5 physicians (5)
- Hospital based practice (6)
- Academic practice (7)
- Private equity group practice (8)

Display This Question:

If For my 3rd leave, my employer/benefits were the same as the prior leave. (same pay circumstances,... = No

Q355 Third family/maternity leave: Did your practice/workplace have a maternity leave policy in place? (not including FMLA)

- Yes (1)
- No (2)
- Unsure (3)

Display This Question:

If For my 3rd leave, my employer/benefits were the same as the prior leave. (same pay circumstances,... = No

Q356 Third family/maternity leave: Were you provided with adequate information about family and maternity leave, and was the leave policy at your workplace easy to understand?

- Extremely inadequate (1)
- Somewhat inadequate (2)
- Neither adequate nor inadequate (3)
- Somewhat adequate (4)
- Extremely adequate (5)

Display This Question:

If For my 3rd leave, my employer/benefits were the same as the prior leave. (same pay circumstances,... = No

Q357 Third family/maternity leave: How many weeks were available to you?

________________________________________________________________

Q358 Third family/maternity leave: How many weeks did you take off?

________________________________________________________________

Q359 Third family/maternity leave: How many weeks of leave would you have liked to receive?

________________________________________________________________

Q360 Third family/maternity leave: Source of leave days: (you may select more than one)

- Sick leave (1)
- Vacation days (2)
- Paid time off (3)
- Specified maternity leave days (4)
- Other (5) __________________________________________________

Display This Question:

If For my 3rd leave, my employer/benefits were the same as the prior leave. (same pay circumstances,... = No

Q361 Third family/maternity leave: Regarding pay during leave, which describes your situation best?

- I did not receive any pay during leave (1)
- I received partial pay during leave (2)
- I received full pay during leave (3)
- Unable to recall (4)
- Other (5)

Display This Question:

If Third family/maternity leave: Regarding pay during leave, which describes your situation best? = I received partial pay during leave

Q362 Third family/maternity leave: How many weeks were paid?

________________________________________________________________

Display This Question:

If Third family/maternity leave: Regarding pay during leave, which describes your situation best? = I did not receive any pay during leave

Or Third family/maternity leave: Regarding pay during leave, which describes your situation best? = I received partial pay during leave

Or Third family/maternity leave: Regarding pay during leave, which describes your situation best? = Unable to recall

Or Third family/maternity leave: Regarding pay during leave, which describes your situation best? = Other

Q363 Third family/maternity leave: Did you experience any financial hardship during maternity leave due to change in compensation?

- Yes (1)
- No (2)
- Not applicable (3)
- I cannot recall (4)

Q364 Third family/maternity leave: If applicable, did you experience a sizable loss in your clinical production bonus during leave?

- Yes (1)
- No (2)
- Not applicable (3)

Q365 Third family/maternity leave: Upon return from maternity/family leave did you experience a reduction in hours worked per week compared to that prior to leave?

- Yes (fill in approx new hours worked below) (4) __________________________________________________
- No (5)
- Not applicable (6)

Display This Question:

If Third family/maternity leave: Upon return from maternity/family leave did you experience a reduct... = Yes (fill in approx new hours worked below)

Q366 Third family/maternity leave: If applicable, why did you work less hours/week after your family/maternity leave?

- Personal choice (1)
- Determined by employer (2)
- Other (4)
- Not applicable (5)

Q367 Third family/maternity leave: Regarding breast/chestfeeding, which best describes your situation:

- I chose to breast/chestfeed at some point in my postpartum period (inclusive or exclusive) (1)
- I wanted to breast/chestfeed but was unable to (2)
- I chose not to breast/chestfeed (3)
- I prefer not to answer (4)

Display This Question:

If Third family/maternity leave: Regarding breast/chestfeeding, which best describes your situation: = I chose to breast/chestfeed at some point in my postpartum period (inclusive or exclusive)

Q368 Third family/maternity leave: If you chose to breastfeed/chestfeed, were you accommodated by your employer to allow adequate time for milk expression?

- Extremely inadequate (1)
- Moderately inadequate (2)
- Slightly inadequate (3)
- Neither adequate nor inadequate (4)
- Slightly adequate (5)
- Moderately adequate (6)
- Extremely adequate (7)
- Not applicable (8)

Display This Question:

If Third family/maternity leave: Regarding breast/chestfeeding, which best describes your situation: = I chose to breast/chestfeed at some point in my postpartum period (inclusive or exclusive)

Q369 Third family/maternity leave: Were you made to feel guilty or penalized for time missed for milk expression?

- Yes (1)
- Maybe (2)
- No (3)
- Not applicable (4)

Display This Question:

If Third family/maternity leave: Regarding breast/chestfeeding, which best describes your situation: = I chose to breast/chestfeed at some point in my postpartum period (inclusive or exclusive)

Q370 Third family/maternity leave: If applicable, do you feel your job impacted the duration of your breastfeeding in a negative way?

- No (1)
- Maybe (2)
- Yes (3)
- Not applicable (4)

Q371 Third family/maternity leave: Did you feel supported by colleagues and administration during maternity leave and upon return to work?

- Definitely yes (1)
- Probably yes (2)
- Probably not (3)
- Definitely not (4)

Q372 Third family/maternity leave: Did you consider leaving your job after the birth of your child?

- Yes (1)
- No (2)
- I did leave my job (3)
- Unable to recall (4)

Q373 Third family/maternity leave: Did you suffer with post-partum depression, anxiety, obsessive compulsive disorder or other mental health disorders in the post-partum period?

- Yes (1)
- Maybe (2)
- No (3)

Q374 Third family/maternity leave: What option did you pursue for childcare for the majority of the first year of life?

- Nanny/home childcare (1)
- Relative or family (2)
- Daycare facility (3)
- Stay at home spouse (4)
- I stayed at home to watch my child (5)
- Other (6) __________________________________________________

Q375 Third family/maternity leave: Did you feel a greater sense of burnout upon return to work?

- Yes (1)
- Maybe (2)
- No (3)

Q376 Third family/maternity leave: Select all that apply: I experienced...

- Derogatory comments by colleagues or staff (1)
- Financial pressure from coworkers / institution (2)
- Termination from my position (3)
- Delay in partnership due to leave (4)
- Anger from patients regarding rescheduled appointments (5)
- Continued pressure to answer emails / perform ancillary duties (6)
- Continued expectation to participate in academic endeavors i.e. submit manuscripts, mentor trainees (7)
- Pressure to return to work earlier than planned (8)
- Other (9) __________________________________________________

Q377 Did the circumstances of this leave impact your future family planning?

- Definitely yes (1)
- Probably yes (2)
- Might or might not (3)
- Probably not (4)
- Definitely not (5)

Q378 Third family/maternity leave: Did your partner take family leave?

- Yes (1)
- No (2)
- Not applicable (4)

Q379 Third family/maternity leave: What was your overall level of satisfaction with your family/maternity leave experience?

- Very unsatisfied (1)
- Somewhat unsatisfied (2)
- Neither unsatisfied nor satisfied (3)
- Somewhat satisfied (4)
- Very satisfied (5)

End of Block: Third Child **

Start of Block: Fourth Child **

Q380 In what year did you take your 4th family/maternity leave after medical school?

________________________________________________________________

Q381 How old were you when you took your 4th family/maternity leave after medical school?

________________________________________________________________

Q382 Fourth family/maternity leave: Choose option that best applies

- Pregnancy with no intervention (1)
- Intervention assisted pregnancy (2)
- Adoption (3)

Display This Question:

If Fourth family/maternity leave: Choose option that best applies = Intervention assisted pregnancy

Q383 If your pregnancy required fertility interventions, did you receive adequate support for time off from work for appointments/procedures?

- Extremely inadequate time off/support (1)
- Somewhat inadequate time off/support (2)
- Neutral (3)
- Mostly adequate time off/support (4)
- Extremely adequate time off/support (5)

Q384 For my fourth leave, my employer/benefits were the same as the prior leave. (same pay circumstances, same number of allowed weeks off)

- Yes (1)
- No (2)

Display This Question:

If For my fourth leave, my employer/benefits were the same as the prior leave. (same pay circumstanc... = No

Q385 Fourth family/maternity leave: Which best describes your practice setting at the time of leave?

- Resident in training (1)
- Fellow in training (2)
- Solo practice (3)
- Private practice ≤5 physicians (4)
- Private practice >5 physicians (5)
- Hospital based practice (6)
- Academic practice (7)
- Private equity group practice (8)

Display This Question:

If For my fourth leave, my employer/benefits were the same as the prior leave. (same pay circumstanc... = No

Q386 Fourth family/maternity leave: Did your practice/workplace have a maternity leave policy in place? (not including FMLA)

- Yes (1)
- No (2)
- Unsure (3)

Display This Question:

If For my fourth leave, my employer/benefits were the same as the prior leave. (same pay circumstanc... = No

Q387 Fourth family/maternity leave: Were you provided with adequate information about family and maternity leave, and was the leave policy at your workplace easy to understand?

- Extremely inadequate (1)
- Somewhat inadequate (2)
- Neither adequate nor inadequate (3)
- Somewhat adequate (4)
- Extremely adequate (5)

Display This Question:

If For my fourth leave, my employer/benefits were the same as the prior leave. (same pay circumstanc... = No

Q388 Fourth family/maternity leave: How many weeks were available to you?

________________________________________________________________

Q389 Fourth family/maternity leave: How many weeks did you take off?

________________________________________________________________

Q390 Fourth family/maternity leave: How many weeks of leave would you have liked to receive?

________________________________________________________________

Q391 Fourth family/maternity leave: Source of leave days: (you may select more than one)

- Sick leave (1)
- Vacation days (2)
- Paid time off (3)
- Specified maternity leave days (4)
- Other (5) __________________________________________________

Display This Question:

If For my fourth leave, my employer/benefits were the same as the prior leave. (same pay circumstanc... = No

Q392 Fourth family/maternity leave: Regarding pay during leave, which describes your situation best?

- I did not receive any pay during leave (1)
- I received partial pay during leave (2)
- I received full pay during leave (3)
- Unable to recall (4)
- Other (5)

Display This Question:

If Fourth family/maternity leave: Regarding pay during leave, which describes your situation best? = I received partial pay during leave

Q393 Fourth family/maternity leave: How many weeks were paid?

________________________________________________________________

Display This Question:

If Fourth family/maternity leave: Regarding pay during leave, which describes your situation best? = I did not receive any pay during leave

Or Fourth family/maternity leave: Regarding pay during leave, which describes your situation best? = I received partial pay during leave

Or Fourth family/maternity leave: Regarding pay during leave, which describes your situation best? = Unable to recall

Or Fourth family/maternity leave: Regarding pay during leave, which describes your situation best? = Other

Q394 Fourth family/maternity leave: Did you experience any financial hardship during maternity leave due to change in compensation?

- Yes (1)
- No (2)
- Not applicable (3)
- I cannot recall (4)

Q395 Fourth family/maternity leave: If applicable, did you experience a sizable loss in your clinical production bonus during leave?

- Yes (1)
- No (2)
- Not applicable (3)

Q396 Fourth family/maternity leave: Upon return from maternity/family leave did you experience a reduction in hours worked per week compared to that prior to leave?

- Yes (fill in approx new hours worked below) (4) __________________________________________________
- No (5)
- Not applicable (6)

Display This Question:

If Fourth family/maternity leave: Upon return from maternity/family leave did you experience a reduc... = Yes (fill in approx new hours worked below)

Q397 Fourth family/maternity leave: If applicable, why did you work less hours/week after your family/maternity leave?

- Personal choice (1)
- Determined by employer (2)
- Other (4)
- Not applicable (5)

Q398 Fourth family/maternity leave: Regarding breast/chestfeeding, which best describes your situation:

- I chose to breast/chestfeed at some point in my postpartum period (inclusive or exclusive) (1)
- I wanted to breast/chestfeed but was unable to (2)
- I chose not to breast/chestfeed (3)
- I prefer not to answer (4)

Display This Question:

If Fourth family/maternity leave: Regarding breast/chestfeeding, which best describes your situation: = I chose to breast/chestfeed at some point in my postpartum period (inclusive or exclusive)

Q399 Fourth family/maternity leave: If you chose to breastfeed/chestfeed, were you accommodated by your employer to allow adequate time for milk expression?

- Extremely inadequate (1)
- Moderately inadequate (2)
- Slightly inadequate (3)
- Neither adequate nor inadequate (4)
- Slightly adequate (5)
- Moderately adequate (6)
- Extremely adequate (7)
- Not applicable (8)

Display This Question:

If Fourth family/maternity leave: Regarding breast/chestfeeding, which best describes your situation: = I chose to breast/chestfeed at some point in my postpartum period (inclusive or exclusive)

Q400 Fourth family/maternity leave: Were you made to feel guilty or penalized for time missed for milk expression?

- Yes (1)
- Maybe (2)
- No (3)
- Not applicable (4)

Display This Question:

If Fourth family/maternity leave: Regarding breast/chestfeeding, which best describes your situation: = I chose to breast/chestfeed at some point in my postpartum period (inclusive or exclusive)

Q401 Fourth family/maternity leave: If applicable, do you feel your job impacted the duration of your breastfeeding in a negative way?

- No (1)
- Maybe (2)
- Yes (3)
- Not applicable (4)

Q402 Fourth family/maternity leave: Did you feel supported by colleagues and administration during maternity leave and upon return to work?

- Definitely yes (1)
- Probably yes (2)
- Probably not (3)
- Definitely not (4)

Q403 Fourth family/maternity leave: Did you consider leaving your job after the birth of your child?

- Yes (1)
- No (2)
- I did leave my job (3)
- Unable to recall (4)

Q404 Fourth family/maternity leave: Did you suffer with postpartum depression, anxiety, obsessive compulsive disorder or other mental health disorders in the postpartum period?

- Yes (1)
- Maybe (2)
- No (3)

Q405 Fourth family/maternity leave: What option did you pursue for childcare for the majority of the first year of life?

- Nanny/home childcare (1)
- Relative or family (2)
- Daycare facility (3)
- Stay at home spouse (4)
- I stayed at home to watch my child (5)
- Other (6) __________________________________________________

Q406 Fourth family/maternity leave: Did you feel a greater sense of burnout upon return to work?

- Yes (1)
- Maybe (2)
- No (3)

Q407 Fourth family/maternity leave: Select all that apply: I experienced...

- Derogatory comments by colleagues or staff (1)
- Financial pressure from coworkers / institution (2)
- Termination from my position (3)
- Delay in partnership due to leave (4)
- Anger from patients regarding rescheduled appointments (5)
- Continued pressure to answer emails / perform ancillary duties (6)
- Continued expectation to participate in academic endeavors i.e. submit manuscripts, mentor trainees (7)
- Pressure to return to work earlier than planned (8)
- Other (9) __________________________________________________

Q408 Did the circumstances of this leave impact your future family planning?

- Definitely yes (1)
- Probably yes (2)
- Might or might not (3)
- Probably not (4)
- Definitely not (5)

Q409 Fourth family/maternity leave: Did your partner take family leave?

- Yes (1)
- No (2)
- Not applicable (4)

Q410 Fourth family/maternity leave: What was your overall level of satisfaction with your family/maternity leave experience?

- Very unsatisfied (1)
- Somewhat unsatisfied (2)
- Neither unsatisfied nor satisfied (3)
- Somewhat satisfied (4)
- Very satisfied (5)

End of Block: Fourth Child **

Start of Block: Fifth Child **

Q411 In what year did you take your 5th family/maternity leave after medical school?

________________________________________________________________

Q412 How old were you when you took your 5th family/maternity leave after medical school?

________________________________________________________________

Q413 Fifth family/maternity leave: Choose option that best applies

- Pregnancy with no intervention (1)
- Intervention assisted pregnancy (2)
- Adoption (3)

Display This Question:

If Fifth family/maternity leave: Choose option that best applies = Intervention assisted pregnancy

Q414 If your pregnancy required fertility interventions, did you receive adequate support for time off from work for appointments/procedures?

- Extremely inadequate time off/support (1)
- Somewhat inadequate time off/support (2)
- Neutral (3)
- Mostly adequate time off/support (4)
- Extremely adequate time off/support (5)

Q415 For my fifth leave, my employer/benefits were the same as the prior leave. (same pay circumstances, same number of allowed weeks off)

- Yes (1)
- No (2)

Display This Question:

If For my fifth leave, my employer/benefits were the same as the prior leave. (same pay circumstance... = No

Q416 Fifth family/maternity leave: Which best describes your practice setting at the time of leave?

- Resident in training (1)
- Fellow in training (2)
- Solo practice (3)
- Private practice ≤5 physicians (4)
- Private practice >5 physicians (5)
- Hospital based practice (6)
- Academic practice (7)
- Private equity group practice (8)

Display This Question:

If For my fifth leave, my employer/benefits were the same as the prior leave. (same pay circumstance... = No

Q417 Fifth family/maternity leave: Did your practice/workplace have a maternity leave policy in place? (not including FMLA)

- Yes (1)
- No (2)
- Unsure (3)

Display This Question:

If For my fifth leave, my employer/benefits were the same as the prior leave. (same pay circumstance... = No

Q418 Fifth family/maternity leave: Were you provided with adequate information about family and maternity leave, and was the leave policy at your workplace easy to understand?

- Extremely inadequate (1)
- Somewhat inadequate (2)
- Neither adequate nor inadequate (3)
- Somewhat adequate (4)
- Extremely adequate (5)

Display This Question:

If For my fifth leave, my employer/benefits were the same as the prior leave. (same pay circumstance... = No

Q419 Fifth family/maternity leave: How many weeks were available to you?

________________________________________________________________

Q420 Fifth family/maternity leave: How many weeks did you take off?

________________________________________________________________

Q421 Fifth family/maternity leave: How many weeks of leave would you have liked to receive?

________________________________________________________________

Q422 Fifth family/maternity leave: Source of leave days: (you may select more than one)

- Sick leave (1)
- Vacation days (2)
- Paid time off (3)
- Specified maternity leave days (4)
- Other (5) __________________________________________________

Display This Question:

If For my fifth leave, my employer/benefits were the same as the prior leave. (same pay circumstance... = No

Q423 Fifth family/maternity leave: Regarding pay during leave, which describes your situation best?

- I did not receive any pay during leave (1)
- I received partial pay during leave (2)
- I received full pay during leave (3)
- Unable to recall (4)
- Other (5)

Display This Question:

If Fifth family/maternity leave: Regarding pay during leave, which describes your situation best? = I received partial pay during leave

Q424 Fifth family/maternity leave: How many weeks were paid?

________________________________________________________________

Display This Question:

If Fifth family/maternity leave: Regarding pay during leave, which describes your situation best? = I did not receive any pay during leave

Or Fifth family/maternity leave: Regarding pay during leave, which describes your situation best? = I received partial pay during leave

Or Fifth family/maternity leave: Regarding pay during leave, which describes your situation best? = Unable to recall

Or Fifth family/maternity leave: Regarding pay during leave, which describes your situation best? = Other

Q425 Fifth family/maternity leave: Did you experience any financial hardship during maternity leave due to change in compensation?

- Yes (1)
- No (2)
- Not applicable (3)
- I cannot recall (4)

Q426 Fifth family/maternity leave: If applicable, did you experience a sizable loss in your clinical production bonus during leave?

- Yes (1)
- No (2)
- Not applicable (3)

Q427 Fifth family/maternity leave: Upon return from maternity/family leave did you experience a reduction in hours worked per week compared to that prior to leave?

- Yes (fill in approx new hours worked below) (4) __________________________________________________
- No (5)
- Not applicable (6)

Display This Question:

If Fifth family/maternity leave: Upon return from maternity/family leave did you experience a reduct... = Yes (fill in approx new hours worked below)

Q428 Fifth family/maternity leave: If applicable, why did you work less hours/week after your family/maternity leave?

- Personal choice (1)
- Determined by employer (2)
- Other (4)
- Not applicable (5)

Q429 Fifth family/maternity leave: Regarding breast/chestfeeding, which best describes your situation:

- I chose to breast/chestfeed at some point in my postpartum period (inclusive or exclusive) (1)
- I wanted to breast/chestfeed but was unable to (2)
- I chose not to breast/chestfeed (3)
- I prefer not to answer (4)

Display This Question:

If Fifth family/maternity leave: Regarding breast/chestfeeding, which best describes your situation: = I chose to breast/chestfeed at some point in my postpartum period (inclusive or exclusive)

Q430 Fifth family/maternity leave: If you chose to breastfeed/chestfeed, were you accommodated by your employer to allow adequate time for milk expression?

- Extremely inadequate (1)
- Moderately inadequate (2)
- Slightly inadequate (3)
- Neither adequate nor inadequate (4)
- Slightly adequate (5)
- Moderately adequate (6)
- Extremely adequate (7)
- Not applicable (8)

Display This Question:

If Fifth family/maternity leave: Regarding breast/chestfeeding, which best describes your situation: = I chose to breast/chestfeed at some point in my postpartum period (inclusive or exclusive)

Q431 Fifth family/maternity leave: Were you made to feel guilty or penalized for time missed for milk expression?

- Yes (1)
- Maybe (2)
- No (3)
- Not applicable (4)

Display This Question:

If Fifth family/maternity leave: Regarding breast/chestfeeding, which best describes your situation: = I chose to breast/chestfeed at some point in my postpartum period (inclusive or exclusive)

Q432 Fifth family/maternity leave: If applicable, do you feel your job impacted the duration of your breastfeeding in a negative way?

- No (1)
- Maybe (2)
- Yes (3)
- Not applicable (4)

Q433 Fifth family/maternity leave: Did you feel supported by colleagues and administration during maternity leave and upon return to work?

- Definitely yes (1)
- Probably yes (2)
- Probably not (3)
- Definitely not (4)

Q434 Fifth family/maternity leave: Did you consider leaving your job after the birth of your child?

- Yes (1)
- No (2)
- I did leave my job (3)
- Unable to recall (4)

Q435 Fifth family/maternity leave: Did you suffer with post-partum depression, anxiety, obsessive compulsive disorder or other mental health disorders in the post-partum period?

- Yes (1)
- Maybe (2)
- No (3)

Q436 Fifth family/maternity leave: What option did you pursue for childcare for the majority of the first year of life?

- Nanny/home childcare (1)
- Relative or family (2)
- Daycare facility (3)
- Stay at home spouse (4)
- I stayed at home to watch my child (5)
- Other (6) __________________________________________________

Q437 Fifth family/maternity leave: Did you feel a greater sense of burnout upon return to work?

- Yes (1)
- Maybe (2)
- No (3)

Q438 Fifth family/maternity leave: Select all that apply: I experienced...

- Derogatory comments by colleagues or staff (1)
- Financial pressure from coworkers / institution (2)
- Termination from my position (3)
- Delay in partnership due to leave (4)
- Anger from patients regarding rescheduled appointments (5)
- Continued pressure to answer emails / perform ancillary duties (6)
- Continued expectation to participate in academic endeavors i.e. submit manuscripts, mentor trainees (7)
- Pressure to return to work earlier than planned (8)
- Other (9) __________________________________________________

Q439 Did the circumstances of this leave impact your future family planning?

- Definitely yes (1)
- Probably yes (2)
- Might or might not (3)
- Probably not (4)
- Definitely not (5)

Q440 Fifth family/maternity leave: Did your partner take family leave?

- Yes (1)
- No (2)
- Not applicable (4)

Q441 Fifth family/maternity leave: What was your overall level of satisfaction with your family/maternity leave experience?

- Very unsatisfied (1)
- Somewhat unsatisfied (2)
- Neither unsatisfied nor satisfied (3)
- Somewhat satisfied (4)
- Very satisfied (5)

End of Block: Fifth Child **
